# Prospective Evaluation of Kidney Function in Long-Term Survivors of Pediatric CNS Tumors

**DOI:** 10.3390/curroncol29080421

**Published:** 2022-07-28

**Authors:** Natalia Stepien, Viktoria Handler, Johannes Gojo, Amedeo A. Azizi, Lisa Mayr, Chryssa Grylli, Daniela Schwarz, Monika Chocholous, Irene Slavc, Michael Boehm, Andreas Peyrl

**Affiliations:** 1 Division of Neonatology, Pediatric Intensive Care and Neuropediatrics, Department of Pediatrics and Adolescent Medicine, Comprehensive Center for Pediatrics, Medical University Vienna, 1090 Vienna, Austria; natalia.stepien@meduniwien.ac.at (N.S.); johannes.gojo@meduniwien.ac.at (J.G.); amedeo.azizi@meduniwien.ac.at (A.A.A.); lisa.mayr@meduniwien.ac.at (L.M.); schwarzdani@gmx.at (D.S.); monika.chocholous@meduniwien.ac.at (M.C.); irene.slavc@meduniwien.ac.at (I.S.); 2 Department of Gynecology and Obstetrics, KRAGES Oberpullendorf, 7350 Oberpullendorf, Austria; viktoria.handler@krages.at; 3 Division of Pediatric Pulmonology, Allergology, and Endocrinology, Department of Pediatrics and Adolescent Medicine, Medical University of Vienna, 1090 Vienna, Austria; chryssa.grylli@meduniwien.ac.at; 4 Division of Pediatric Nephrology and Gastroenterology, Comprehensive Center for Pediatrics, Medical University of Vienna, 1090 Vienna, Austria; michael.boehm@meduniwien.ac.at

**Keywords:** renal function, glomerular filtration rate, Fanconi syndrome, chronic kidney disease, childhood cancer survivors, supportive care

## Abstract

Purpose: Numerous acute effects of chemotherapeutics on kidney function are well described. However, data on the long-term effects of chemotherapy in the growing population of childhood central nervous system (CNS) tumor survivors is limited. We aimed to evaluate the kidney function of a cohort of long-term CNS tumor survivors treated with different standard chemotherapeutic regimens. Methods: Patients treated for a CNS tumor were prospectively evaluated up to 12 years after completion of their therapy. Examination of kidney function was performed during routine follow-up visits. Blood pressure and blood and urine parameters were analyzed for kidney function evaluation. Glomerular function was assessed by calculating the estimated glomerular filtration rate (eGFR), tubular functions were analyzed by measuring serum electrolytes, bicarbonate and phosphate reabsorption, and proteinuria was assessed by calculating the protein/creatinine ratio and phosphate reabsorption. Results: None of the 65 patients evaluated suffered from clinically relevant kidney impairment (eGFR < 90 mL/min/L, 73 m^2^). There was no association between chemotherapy dose and eGFR. Only two patients showed mild signs of tubulopathy and 11 patients were diagnosed with elevated blood pressure. Conclusion: With adequate supportive measures, such as sufficient hydration according to chemotherapy protocol guidelines, as well as avoidance or close monitoring of additional nephrotoxic medication, impaired kidney function is rare in CNS tumor survivors treated with standard chemotherapy. Nonetheless, long-term follow-up is essential for early detection of mild impairment of kidney function.

## 1. Introduction

CNS tumors constitute the most common solid malignancy in the pediatric population with an incidence rate of 2–4/100,000. They are comprised of more than hundred different entities with very divergent biological behavior [1]. Treatment may include observation by MRI, surgery, chemotherapy, radiation or a combination of the previously mentioned, and current studies focus on risk stratification for the prevention of therapy associated side effects [2,3,4]. Generally, over the last several decades, survival of pediatric central nervous system (CNS) tumor patients has improved significantly [5]. Unfortunately, cure often comes at a cost for these patients due to induction of various late effects, necessitating accurate assessment of sequelae for early detection and possible preventive measures. In a study on chronic health conditions in adult survivors of childhood cancer, CNS tumor survivors were found to be among the population at highest risk for developing a severe health condition [6]. When focusing on the kidney function in this patient population, certain subgroups have been found to harbor an increased risk for long-term nephrotoxicity [7]. However, data on the chemotherapy protocols applied were not available in this study, and the diagnosis of kidney disease was derived from a registry, which might underestimate the presence of slightly reduced kidney function [7]. The retrospective analysis of the Childhood Cancer Survivor Study revealed a significantly higher risk for late-onset kidney disease in the patient population when compared to their siblings. The main drawback of this study is that kidney disease was self-reported by the patients and therefore mild impairment would not have been recognized [8]. A recent Cochrane analysis evaluating the adverse effects of chemotherapy on kidney function in the pediatric population highlighted the difficulties of different reporting methods for kidney function, and the complexity of an accurate assessment for each cytostatic agent due to the broad variability of entities and respective chemotherapy protocols [9]. Indisputably, cytostatic drugs may lead to acute kidney injury, particularly if administered without adequate supportive measures.

The major chemotherapeutics that impair kidney function are platinum compounds (i.e., cisplatin, carboplatin), alkylating agents (i.e., ifosfamide), and the antifolate agent methotrexate (MTX), which are all included in routine treatment protocols for childhood CNS tumors [10]. Nephrotoxicity of chemotherapeutic medication may manifest as acute kidney injury, but also as long-term kidney dysfunction [11].

The aim of this study was to determine the prevalence and severity of chemotherapy-induced nephrotoxicity in the specific group of patients following treatment for a CNS tumor in childhood or adolescence.

## 2. Patients and Methods

### 2.1. Patients

Patients included in the study were treated for a CNS tumor at the Department of Pediatrics and Adolescent Health, Medical University of Vienna. 

Between 1999 and 2018, 279 children received chemotherapy for their primary CNS tumor at our institution, and 123 of these patients deceased. Of the 156 children that achieved long-term survival, 19 patients were treated for a recurrent tumor following initial chemotherapy, and 31 patients were lost to follow-up. In total, 65 of potentially 106 eligible patients agreed to participate and were enrolled in the study and prospectively investigated for their kidney function (Figure S1). Data on tumor histology and treatment was retrieved from the patient’s charts. The 65 participants did not differ significantly from the 41 potentially eligible nonparticipants in the distribution of sex, diagnosis, or chemotherapy received.

Before each initiation of a chemotherapy cycle, all patients were screened for eGFR as a standard procedure. During their chemotherapy, all patients were hydrated sufficiently according to their body surface area (BSA) as recommended in their treatment protocols (2400–3000 mL/m^2^/d of prespecified solution). Diuretics were used as defined in their treatment protocol (i.e., mannitol for platinum compounds and furosemide for other chemotherapeutic agents), and blood levels of additional nephrotoxic medication (i.e., vancomycin, certomycin, gentamicin, amphotericin) were closely monitored [12]. All patients had completed their treatment at least one year before kidney evaluation. The time of examination was defined by the patients’ routine follow-up protocol.

### 2.2. Methods

Prospective evaluation of kidney function included anthropometric parameters (height and weight, body mass index (BMI)) and blood pressure measurement. For each parameter the standard deviation score (SDS) was evaluated. Hypertension was evaluated according to the NCI CTCAE criteria 5.0 [13,14]. The original Schwartz formula was used to calculate the estimated glomerular filtration rate. Serum creatinine was measured by the Jaffe method [15]. The cut-off for impaired glomerular function was 90 mL/min/L/1.73 m^2^ [9]. Additional routine laboratory investigations included the following parameters (cut-off values in brackets, if not stated differently, as recommended by the institutional laboratory): serum phosphate (age adjusted [16]), serum magnesium (0.61–1.23 mmol/L), serum bicarbonate (lower limit of normal (LLN): 20 mmol/L), and urinary protein/creatinine ratio (upper limit of normal (ULN): 200 mg/g). Tubular function was further evaluated by calculating the tubular maximum reabsorption of phosphate by GFR (TmP/GFR) (age-adjusted [17]), measurement of glucosuria (ULN 25 mg/dL) and alpha-1-microglobuline (ULN: 20 mg/24 h), and evaluation of electrolyte supplementation.

### 2.3. Statistical Analysis

Continuous variables were described by using median and range. Furthermore, standard deviation scores (SDS) were calculated for length, BMI, and blood pressure. For each chemotherapeutic agent, dose ranges (low/middle/high or low/high—Table S1) were established, considering the target values of the applied protocols (Table S2) and cut-off values defined by previous studies [18,19,20]. Additionally, patients were classified according to their treatment protocols or a combination of protocols. The Kruskal–Wallis test and the Mann–Whitney U test were used to determine if different chemotherapy doses or protocols resulted in altered renal parameters (eGFR). The latter one was used in cases in which only two groups were compared.

Statistical analysis was performed with the IBM SPSS Statistics for Windows, version 28.0.1.0 (IBM Corp., Armonk, NY, USA). Each test result was considered as significant when the two-sided *p*-value was less than 0.05.

The study protocol was approved by the Ethics Committee of the Medical University of Vienna (EK 520/2009), and a written informed consent was obtained from all study participants and/or their parents.

## 3. Results

### 3.1. Patients’ Characteristics and CNS Tumor Entities

Among the 65 patients enclosed, 44 children were male (68%) and 21 female (32%, M:F ratio 2:1), and the overall median age at time of diagnosis was 4.7 years (range 0.0–20.1 years). Chemotherapy started at a median age of 4.8 years (range 0.1–20.1 years). The median patient age at the time of follow-up examination was 12.7 years (range 3.2–25.5 years) and the median time between the end of chemotherapy and follow-up was 3.2 years (range 1.0–12.3 years). Diagnoses were low-grade glioma (n = 15), medulloblastoma (n = 14), high-grade glioma (n = 10), ependymoma (n = 8), CNS germ cell tumor (n = 6), atypical teratoid rhabdoid tumor (n = 4), CNS PNET (n = 3; two of them were reclassified after the WHO 2016 classification: one high-grade neuroepithelial tumor and one CNS NB-Foxr2), pineoblastoma (n = 2), choroid plexus carcinoma (n = 1), epitheloid sarcoma (n = 1), and one diffuse leptomeningeal glioneuronal tumor (n = 1). Localization of tumors is represented in Figure S2.

### 3.2. Chemotherapy and Additional Nephrotoxic Agents

All patients received chemotherapy according to standard treatment protocols (HIT-91 [21], HIT-2000 [22], HIT-GBM-C [23], HIT-HGG-2007 [24], SIOP-GCT-96 [25], SIOP-LGG-2004 [26], and MUV-ATRT [27]. Five patients received high-dose chemotherapy with stem cell support [28]. Cumulative dose and dose range of each administered chemotherapeutic agent and the number of patients are listed in Table 1. Chemotherapeutic agents and cumulative doses for each protocol are listed in Table S2. Hyperhydration was performed according to each protocol with a prespecified solution, by using 2400–3000 mL/m^2^, starting at least 12 h before start of chemotherapy, until at least 12 h after the end of chemotherapy.

At least one of the chemotherapeutic agents with the highest risk of nephrotoxicity (cisplatin, carboplatin, ifosfamide, and methotrexate) was administered in 58 patients (89%). All patients receiving ifosfamide or cyclophosphamide were treated with the cytoprotective adjuvant mesna. None of the patients suffered from acute kidney injury according to CTCAE 5.0 during the time of treatment as assessed by chart review [14]. In total, 19 patients (29%) received craniospinal irradiation, and two patients (3%) received spinal irradiation. Among these patients, 20 (95%) were treated with chemotherapeutic agents expected to be highly nephrotoxic. Additionally, 18 patients (28%) received intraventricular chemotherapy.

At least one additional nephrotoxic medication, including vancomycin, certomycin, gentamicin and amphotericin, had been applied in 38 patients (58%) during the course of their treatment. Among this group, 14 patients (37%) received nephrotoxic medication concomitant to chemotherapy.

### 3.3. Glomerular and Tubular Function

The glomerular function was not affected after the administration of the above-described chemotherapy. Median eGFR was 150.1.0 mL/min/1.73 m^2^ (range 96.5–253.1 mL/min/1.73 m^2^). Patients who had received at least two chemotherapeutic agents classified as particularly nephrotoxic had a median eGFR of 150.1 mL/min/1.73 m^2^ (range 96.5–253.1 mL/min/1.73 m^2^). By using the original Schwartz formula, none of the patients evaluated had an eGFR below 90 mL/min/1.73 m^2^. There was no significant association between the cumulative dose of any cytostatic agent and eGFR (Table 2). Neither the chemotherapy protocol applied, nor the administration of more than two particularly nephrotoxic cytostatic agents (carboplatin, cisplatin, ifosfamide, and methotrexate) showed a significant difference in eGFR (Figure S3). There was no significant effect of nephrotoxic medication (gentamycin, certomycin, vancomycin, and amphotericin) on eGFR (Table 2).

A total of 23 patients presented with a singular event of elevated blood pressure (systolic value between 120 and 140 mmHg or diastolic value > 79 mmHg). These values were reproducible at follow-up (following office visit) in only 11 patients; one patient was lost to follow up, and 11 patients had blood pressure values within the normal range. One patient was diagnosed with a midaortic syndrome, a secondary cause other than cancer treatment (Table S3, patient 2). Of the other hypertensive patients, six reached adult age at last follow-up with systolic blood pressure values ranging from 120–133 mmHg, corresponding to CTCAE °1. Patient characteristics, including administered chemotherapeutic regimens of the remaining four pediatric patients are displayed in Table S3.

Of the 65 evaluated patients, seven showed an elevated protein/creatinine ratio >200 mg/g (range 204–311 mg/g). One patient, with a protein/creatinine ratio of 300 mg/g, presented also with increased total protein, glucose, albumin, and alpha-1-microglobulin excretion in the 24-h urine specimen. SDS electrophoresis revealed tubular proteinuria. Another one had an elevated protein/creatinine ratio, albumin/creatinine ratio and a reduction in TmP/GFR. The other four patients presented with normal protein levels in their respective 24-h urine specimens and without further signs of tubulopathy. None of the five remaining patients with a reduction in TmP/GFR had further signs of tubulopathy. One patient was diagnosed with isolated glucosuria. For further details on patient characteristics, see Table S3.

None of the patients suffered from hyponatremia, hypokalemia, hypocalcemia, hypomagnesemia, or low bicarbonate levels at follow-up. None of these patients suffered from hypophosphatemia when using age-adjusted phosphate levels [16]. None of the patients required bicarbonate or other electrolyte substitution.

## 4. Discussion

A decreased kidney function as a consequence of intensive chemotherapy for childhood cancer has been published in several studies [9,11,18,29,30,31,32], but data focusing in particular on the long-term nephrotoxicity of chemotherapeutic agents used for the treatment of CNS tumors in childhood is scarce [19,33]. We report on 65 children treated with nephrotoxic chemotherapeutic compounds used most frequently for childhood CNS tumors such as cisplatin, carboplatin, ifosfamide, and methotrexate, all known to be causative of acute renal toxicity. Previous reports on evaluation of their long-term nephrotoxic effect led to divergent results that very much depended on the assessment method of renal function, type of malignancy and additional therapy (i.e., abdominal radiation, nephrectomy) [9,34]. The potential effects of chemotherapeutic agents on renal function vary from mild tubular dysfunction to irreversible renal failure, necessitating dialysis.

The GFR is considered the best overall parameter for kidney function; however, variability of methods used to calculate or estimate GFR is broad [9,35]. In clinical practice, the most ubiquitous method for GFR estimation in the pediatric population is the original Schwartz formula with a cut-off for chronic kidney disease of 90 mL/min/1.73 m^2^. Serum creatinine, height, age, and sex are readily available parameters, making it a very convenient method for GFR estimation in clinical routine. However, a tendency for overestimation of GFR has been shown in several studies [36,37]. Although the Schwartz formula only provides an estimation of GFR, it is often preferred to the cumbersome collection of 24-h urine for creatinine clearance calculation, which is prone to collection errors, especially in children. Furthermore, it is also applicable for young adults, providing a higher accuracy up to the age of 40 years than the Chronic Kidney Disease Epidemiology Collaboration (CKD-EPI) equation [38]. Loss of muscle mass has to be considered when interpreting eGFR in oncology patients. More exact, but also more invasive methods, such as GFR calculation by inulin clearance or alternative filtration markers, are not feasible in a routine screening setting and are preserved for special indications.

In general, the reported incidence of impaired GFR in children treated with chemotherapy varies from 0 to 74% [9]. In our study, eGFR was above 90 mL/min/1.73 m^2^ in all our patients. There was no correlation between the dose of any chemotherapeutic agent or chemotherapy protocol applied and the eGFR (Table 2, Tables S1 and S3). Previously reported risk factors for reduced GFR include nephrectomy, high-dose ifosfamide, cisplatin, carboplatin and high-dose cyclophosphamide [9]. None of the children in our cohort underwent a nephrectomy, explaining in part the lack of impairment in kidney function parameters in our patients.

Regarding the effect of different chemotherapeutic agents on GFR, ifosfamide is known to cause rather tubulopathy than glomerular dysfunction. However, the reported incidence of reduced GFR ranged from 4% to 50% [31,39]. Two prospective studies on this topic reported impaired glomerular function in 22% and 50% of patients after administration of ifosfamide, with a median cumulative dose of 54 g/m^2^ and 106 g/m^2^, respectively [18,31]. Because our patients received a median cumulative ifosfamide dose of 20 g/m^2^ (range 7.5–40.7), our results may support the theory of a dose-dependent long-term effect of ifosfamide on GFR, despite the lack of a statistically significant difference in eGFR between the dosing groups in our study [18,20,34,40].

Cisplatin on its own is also reported to cause long-term reduction in GFR in 29–40% of patients, with median cumulative doses of 528 mg/m^2^ and 500 mg/m^2^, respectively [19,30]. Part of the reported frequency of chronic kidney disease can be attributed to an initial acute kidney injury; however, exact numbers are lacking. Reports that suggest a GFR recovery potential in children [41] as well as in adults [42], are in contrast to reports describing a deterioration of GFR over time, especially in children treated with higher cisplatin doses (>500 mg/m^2^) [34]. Adequate hydration seems to be important to prevent the first hit [29,43,44]. With adequate hydration and mannitol as a diuretic agent according to their respective treatment protocol, none of our patients suffered from an acute kidney injury, and no clinically relevant deterioration of eGFR over time was noted after treatment with a median cumulative cisplatin dose of 300 mg/m^2^.

Carboplatin, a second-generation platinum compound, was only rarely described to have a negative effect on GFR, which is in accordance with our study [45].

The acute nephrotoxicity of methotrexate is indisputable [46], though data on long-term effects are rare. Available data including our own study suggest no further decrease in GFR [11,34].

Despite the use of multiple chemotherapeutic agents for treatment, survivors of CNS tumors in our cohort do not suffer from reduction in GFR. This might be attributed to the comparatively lower dose of each single agent used in protocols for CNS tumor treatment compared to sarcoma protocols.

Despite eGFR being an important overall parameter of renal function, chemotherapeutic agents also have important effects on tubular function, which is not reflected by changes of the eGFR. Tubular dysfunction is characterized by electrolyte disturbances, phosphate and magnesium loss and alpha-1-microglobulinuria. The reported prevalence of hypophosphatemia and hypomagnesemia ranges between 0–37%, and 13–71%, respectively [9,19]. Therapy with ifosfamide, cisplatin, and carboplatin was reported as a risk factor [9,47].

Ifosfamide does not only induce acute tubular toxicity, but may also result in long-term proximal tubular dysfunction, resulting in Fanconi syndrome and consequently rickets and growth retardation [42,48]. Subclinical glucosuria was the most common urinary abnormality finding in 37–88% of patients treated with ifosfamide [18,40]. Glycosuria was only present in one of the patients evaluated in our study. Cisplatin may cause mainly hypomagnesemia, which was reported in 29–71.4% of the patients [19,29]. Carboplatin has similar, but less severe long-term effects [30,49]. The effect of MTX on tubular function seems to be minimal, low-molecular-weight proteinuria has been described, as well as some cases of renal phosphorus loss [11].

Of the 65 patients analyzed in our cohort, only two presented with manifest tubulopathy and one with isolated glucosuria. All three had been treated with at least two of the previously characterized especially nephrotoxic drugs and had received additional nephrotoxic medication. None of our patients received more than 50 g/m^2^ ifosfamide, and the median cumulative dose of cisplatin administered in our study was also lower (300 mg/m^2^) than in the previously mentioned studies [19,29].

A dose dependency of long-term tubular toxicity of ifosfamide was suggested by several authors [20,40,43,50] and is in accordance with the low rate of tubular toxicity reported in this study, where comparatively lower doses of ifosfamide had been used (median cumulative dose 20 g/m^2^ versus 62 g/m^2^ [20]).

An additional important aspect in the evaluation of renal function is blood pressure regulation. Severe systolic hypertension has been reported in 5–15% of patients after treatment with ifosfamide, cisplatin, or high-dose methotrexate [11,19,20,30]. The risk of increased blood pressure was higher in children who were exposed both to cisplatin and cranial irradiation [19]. In our cohort, arterial hypertension was confirmed in 11 patients (17%); however, this arterial hypertension did not require medication, but did support the necessity of regular screening. This is highlighted by the recent report from the Childhood Cancer Survivor Study, which revealed that survivors suffering from diabetes and/or hypertension are a risk population for the development of late-onset renal failure [8].

The contribution of a specific chemotherapeutic agent to the deterioration of any specific renal function parameter is difficult to assess, considering the multi-drug combinations used in most pediatric chemotherapy protocols and the variability of potential effects. The lower doses applied in our cohort of CNS tumor patients when compared to sarcoma patients [18,20], and the lacking need of nephrectomy in contrast to nephroblastoma patients [9] may have contributed to the preservation of kidney function in CNS tumor survivors.

Our study has several limitations. In awareness of the importance of uniform assessment of kidney function, but still considering the difficulty of invasive kidney function measurements in clinical practice, we decided to use the Schwartz formula with the commonest cut-off value of 90 mL/min/1.73 m^2^ for GFR estimation. Because our laboratory uses the Jaffé method for creatinine measurement, we used the original Schwartz formula [15], what might have led to an overestimation of eGFR.

Additionally, the study population is relatively small, thereby making it impossible to divide the cohort into further subgroups or perform multivariate analyses. This would have been of special interest due to the inhomogeneity in the interval between the end of chemotherapy and kidney function investigation, which might neglect other influencing factors, such as a higher susceptibility for a “second hit” event. Further research should focus on risk stratification and prevention methods, thereby focusing on diagnosis and treatment of hypertension.

## 5. Conclusions

Taken together, with adequate supportive measures, such as sufficient hydration according to the respective treatment protocol, as well as avoiding or close monitoring of additional nephrotoxic medication (vancomycin, certomycin, gentamicin, amphotericin), prevalence of long-term nephrotoxicity in CNS tumor is low. To detect mild renal function impairment, long-term follow-up, including basic renal function evaluation (eGFR, blood pressure, blood electrolytes, and urine analysis including protein) is essential.

## Figures and Tables

**Table 1:** Median cumulative dose and dose range for each cytotoxic agent as well as the number of patients treated with each agent.

Chemotherapeutic Agent	Median Cumulative Dose	Dose Range	Number of Patients
Actinomycin	9 mg/m^2^	9–9	1
Bevacizumab	130 mg/kg	110–760	5
Carboplatin	2800 mg/m^2^	600–9350	43
Cisplatin	300 mg/m^2^	63–660	35
Cyclophosphamide	7200 mg/m^2^	800–72,840	28
Doxorubicin	180 mg/m^2^	120–440	5
Etoposide	1650 mg/m^2^	300–3900	39
Ifosfamide	20,341 mg/m^2^	7500–47,200	23
Lomustine	445 mg/m^2^	35–600	15
Methotrexate	25,000 mg/m^2^	5000–30,000	17
Temozolomide	7925 mg/m^2^	2050–18,550	12
Vincristine	15 mg/m^2^	2.6–50.7	47

**Table 2:** *p*-values for the correlation between each chemotherapeutic agent and eGFR, as well as chemotherapy protocol, intensity of chemotherapy, and additional administration of nephrotoxic drugs and eGFR.

Chemotherapeutic Agent Low/Middle/High (l/m/h) or Low/High (l/h)	Correlation with eGFR (*p*-Value)
Carboplatin (l/m/h)	0.869 *
CCNU (l/h)	0.232 °
Cisplatin (l/m/h)	0.912 *
Cyclophosphamide (l/h)	0.469 °
Etoposide (l/m/h)	0.300 *
Ifosfamide (l/m/h)	0.323 *
Methotrexate (l/h)	0.871 °
Temozolomide (l/h)	0.202 °
Vincristine (l/h)	0.189 °
Chemotherapy protocol	0.459 *
Administration of more than 2 nephrotoxic chemotherapeutics	0.830 °
Administration of nephrotoxic supportive medication	0.670 °

* Analysis by Kruskal–Wallis test, ° Analysis by Mann–Whitney *U* test. Two-sided significance level of 0.05.

## Data Availability

The data that support the findings of this study are available from the corresponding author upon reasonable request.

## Supplementary Materials

The following supporting information can be downloaded at: https://www.mdpi.com/article/10.3390/curroncol29080421/s1, Figure S1, flow chart of patient recruitment; Figure S2, Distribution of tumor localization; Figure S3, Correlation of therapy protocols and applied treatment protocols; Table S1, Cut-off levels for chemotherapeutic agents; Table S2, Overview of treatment protocols; Table S3, Patient characteristics of patients with mild renal impairment.
