# Supplementary material for: Prospective Evaluation of Kidney Function in Long-Term Survivors of Pediatric CNS Tumors
Source: Curr Oncol. 2022 Jul 28;29(8):5306–15. doi: 10.3390/curroncol29080421 (PMC9406573; doi:10.3390/curroncol29080421)
Supplement: Supplementary file 1 [file curroncol-29-00421-s001.zip › curroncol-1819853-supplementary.pdf]

Article

Prospective Evaluation of Kidney Function in Long-Term Survivors of Pediatric CNS Tumors

Natalia Stepien, Viktoria Handler, Johannes Gojo, Amedeo A. Azizi, Lisa Mayr, Chryssa Grylli, Daniela Schwarz, Monika Chocholous, Irene Slavc, Michael Boehm and Andreas Peyrl

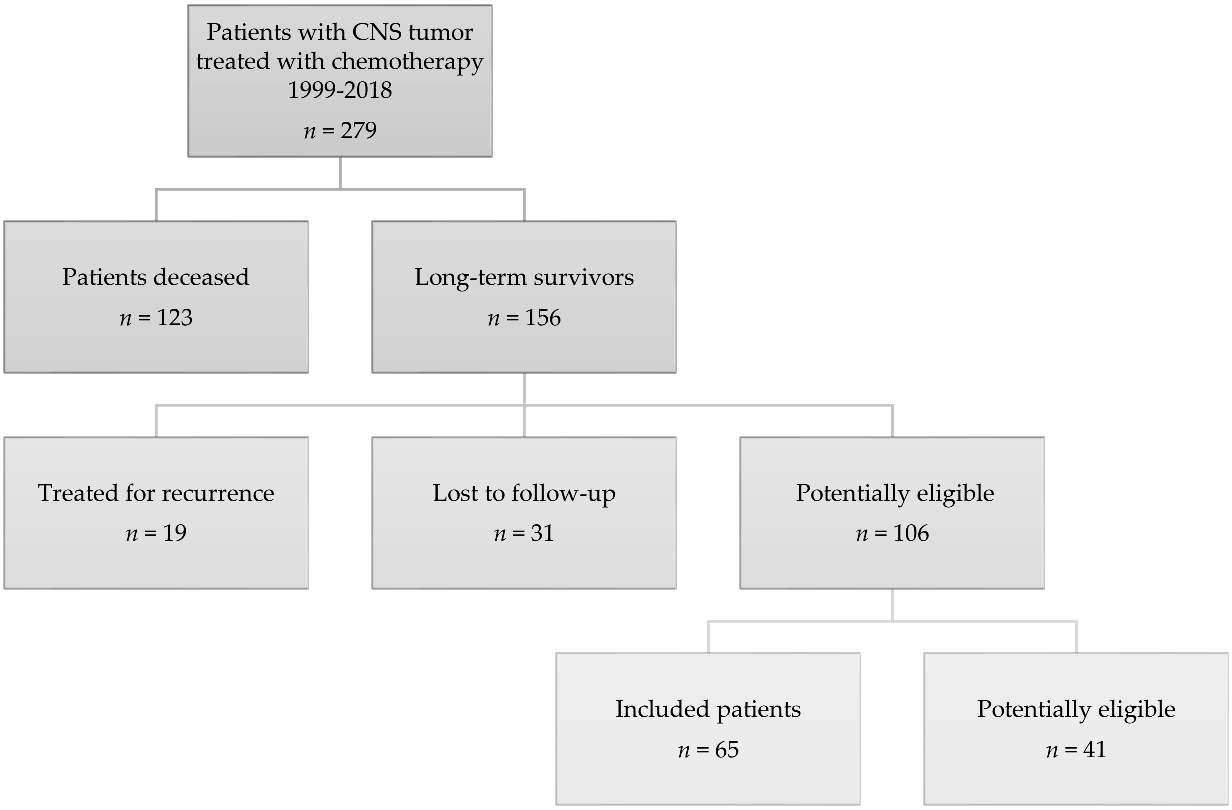

Figure S1. Flow chart of patient recruitment. 41 potentially eligible patients choose not to participate in the study. CNS: central nervous system.

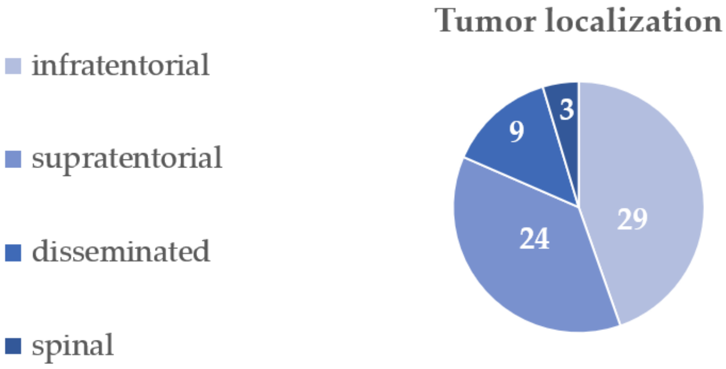

Figure S2. Distribution of tumor localization. Tumor localization was classified into four groups, supratentorial, infratentorial, spinal and disseminated. Tumours were included into the „disseminated" group, if there was visible tumour on the MRI on multiple localizations. CSF positive for tumor cells was not grouped into disseminated. Classification was performed based on the initial presentation. Numbers in pie chart represent absoulte patient numbers.

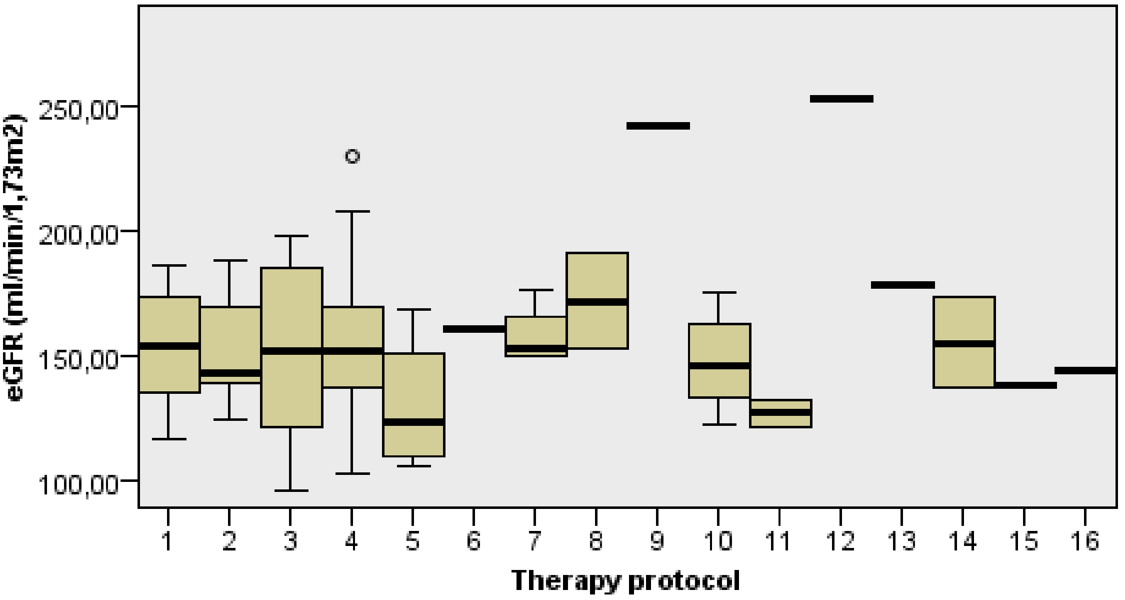

Protocol	*Number*
HIT Maintainance	1
HIT Induction	2
SIOP GCT 96	3
SIOP LGG 2004	4
HIT-CTx + SIOP GCT 96 + Finlay	5
HIT + Finlay	6
HIT + SIOP GCT 96	7
SIOP LGG 2004 + Temozolomide	8
SIOP Ependymoma II	9
Temozolomide	10
PNET 5 MB LR	11
SIOP LGG 2004 + SIOP GCT 96	12
HIT-CTx + SIOP GCT 96	13
SIOP GCT 96 + Temozolomide	14
HIT-CTx + Temozolomide	15
CWS-2002P (High-Risk)	16

Figure 3. Correlation of therapy protocols and applied treatment protocols. Kruskal-Wallis-Test did not show any correlation between therapy protocol applied and eGFR ($p=0.459$). The Table below the gives an overview of therapy combinations patients received for treatment, thereby illustrating the difficulty of further grouping.

Table S1. Cut-off levels for chemotherapeutic agents.

Chemotherapeutic agent	low	middle	high
Carboplatin (mg/m²)	x ≤ 1800 (12)	1800 < x ≤ 3000 (14)	x > 3000 (9)
CCNU (mg/m²)	x ≤ 450 (8)		x > 450 (7)
Cisplatin (mg/m²)	x ≤ 210 (12)	210 < x < 400 (14)	x ≥ 400 (9)
Cyclophosphamide (mg/m²)	x ≤ 4800 (8)		x > 4800 (20)
Etoposide (mg/m²)	x ≤ 1000 (7)	1000 < x ≤ 2250 (24)	x > 2250 (8)
Ifosfamide (mg/m²)	x ≤ 10000 (5)	10000 < x < 30000 (13)	x ≥ 30000 (5)

Methotrexate (mg/m²)	x ≤ 15000 (4)	x > 15000 (13)
Temozolomide (mg/m²)	x < 12000 (7)	x ≥ 120000 (5)
Vincristine (mg/m²)	x ≤ 13,5 (20)	x > 13,5 (27)

Chemotherapeutic agents administered in at least five patients and respective cut-off levels for low/middle/high dose classification as well as number of patients treated within each category. CCNU = Chlorethyl-Cyclohexyl-Nitroso-Urea or Lomustine.

Table S2. Overview of treatment protocols.

**Protocol [Reference]**	**Cumulative dose per m²**
**HIT 91 [14]**	
Cisplatin	800mg
Cytarabine	2400mg
Etoposide	900mg
Ifosfamide	18g
Methotrexate	20g
Lomustine	600mg
Vincristine	36mg
**HIT 2000 [15]**	
Carboplatin	3000mg
Cyclophosphamide	12000mg
Etoposide	2250mg
MTX	20g
Vincristine	49.5mg
Cisplatin	560mg
Lomustine	600mg
**HIT GBM C [16]**	
Cisplatin	800mg
Etoposide	2400mg
Vincristine	16.5mg
Ifosfamide	52.5mg
**HIT HGG 2007**	
Temozolomide	15150mg
**SIOP GCT 96**	
Cisplatin	400mg
Etoposide	1.2g
Ifosfamide	30g
**SIOP LGG 2004**	
Carboplatin	10450mg
Vincristine	76.5mg
**MUV ATRT**	
Cyclophosphamide	7200mg
Cisplatin	300mg
Doxorubicin	180mg
Etoposide	900mg
Ifosfamide	22.5g
Methotrexate	30g
Vincristine	13.5mg
**High-dose chemotherapy with stem cell support**	
Etoposide	1000mg
Carboplatin	2000mg

Thiotepa	1200mg

Prespecified cumulative chemotherapy doses of protocols administered in our cohort. Protocols and doses have been adapted according to each patient's clinical situation. Actual cumulative doses for patients with mild renal impairment are displayed in Table S3.

Table S3. Patient characteristics of patients with mild renal impairment.

Patient number	Age at the time of enrollment (in years)	Sex	Diagnosis	Chemotherapeutic agents and cumulative doses used in the individual patient	Irradiation	Blood pressure (mmHg)	eGFR (ml/min/1,73m²)	Urine examination
**1**	4	m	Germinoma	Carboplatin 2500mg/m² Cisplatin 180mg/m² Cyclophosphamide 6.4g/m² Etoposide 2400mg/m² Ifosfamide 13.5g/m² MTX 10g/m² Thiotepa 900mg/m² VCR 6mg/m²	none	116/53 (SDS 2,36)	107	P/C: 161mg/g A/C: 12mg/g
**2**	4	m	NF1, Opticusglioma, bilateral stenosis of the renal arteries	Carboplatin 9350mg/m² VCR 51mg/m²	none	130/80 (SDS 3,59)	230	P/C: 212mg/g
**3**	9	f	Ependymoma	Carboplatin 1800/m² Cyclophosphamide 7.2g/m² Etoposide 1350mg/m² MTX 20g/m² VCR 9mg/m²	cranial	115/72 (SDS 1,79)	124	P/C: 273mg/g A/C: not evaluable
**4**	4	m	diffuse leptomeningeal glioneuronal tumor	Cisplatin 440mg/m² Etoposide 1500mg/m² Ifosfamide 33000mg/m²	none	111/67 (SDS 1,77)	175	P/C: 204 mg/g A/C: 10mg/g
**5**	7	f	Ependymoma	Carboplatin 300mg/m² Cyclophosphamide 12.0g/m² Etoposide 2250mg/m² MTX 30g/m² VCR 16.5mg/m²	cranial	115/67 (SDS 1,81)	188	P/C 210mg/g
**6**	9	m	ATRT	Adriamycin 440mg/m² Carboplatin 4500mg/m² Cisplatin 175mg/m² Cyclophosphamide 4.6g/m² Etoposide 1334mg/m² Idarubicin 20mg/m² Ifosfamid 16.3g/m² Thiotepa 900mg/m² VCR 4.3mg/m²	cranial	109/62 (SDS 1,24)	134	P/C: 300mg/g A/C: 49mg/g
**7**	7	m	High-grade glioma	Cisplatin 480mg/m² Etoposide 1800mg/m² Ifosfamide 7.5g/m² Temozolomide 3675mg/m²	CNS-axis	89/53 (SDS -1,22)	174	P/C: 311 A/C: 60mg/g
**8**	5	m	Ependymoma	Carboplatin 2800mg/m² Cisplatin 160mg/m² Cyclophosphamide 11.2g/m² Ifosfamide 12.0g/m² Etoposide 2600mg/m² VCR 7,5mg/m²	cranial	86/58 (SDS -1,71)	156	P/C 268 A/C <14
**9**	7	m	ATRT	Carboplatin 600mg/m² Cisplatin 660mg/m² Etoposid 2400mg/m² Ifosfamid 49,5g/m² VCR 1.5mg/m²	cranial	111/64 (SDS 1,62)	104	P/C: 147 A/C: 29 α-1 microglobuline: 26mg/L

Glucose 217mg/dL

Table S3 displays clinical characteristics (diagnosis, pre-treatment co-morbidities, individual chemotherapy doses, type of irradiation, blood pressure) and eGFR of patients with proteinuria as a sign of mild renal impairment. f: female; m: male; NF1: Neurofibromatosis Type I; ATRT: atypical teratoid rhabdoid tumor; MTX: Methotrexate; VCR: Vincristine; SDS: standard deviation score, given for systolic blood pressure; eGFR: estimated glomerular filtration rate, calculated by the original Schwartz formula; P/C: protein/creatinine ratio in mg/g, reference value < 200mg/g; A/C: albumin/creatinine ratio reference value < 30mg/g.
